# Prevalence of Developmental Dyslexia in Primary School Children: A Systematic Review and Meta-Analysis

**DOI:** 10.3390/brainsci12020240

**Published:** 2022-02-10

**Authors:** Liping Yang, Chunbo Li, Xiumei Li, Manman Zhai, Qingqing An, You Zhang, Jing Zhao, Xuchu Weng

**Affiliations:** 1 Key Laboratory of Brain, Cognition and Education Sciences, Ministry of Education, Guangzhou 510631, China; yang-psy@foxmail.com (L.Y.); 2018022967@m.scnu.edu.cn (X.L.); zhaimm@m.scnu.edu.cn (M.Z.); 2019023044@m.scnu.edu.cn (Q.A.); 2019023159@m.scnu.edu.cn (Y.Z.); 2 School of Psychology, South China Normal University, Guangzhou 510631, China; 3 Shanghai Key Laboratory of Psychotic Disorders, Shanghai Mental Health Center, Shanghai Jiao Tong University School of Medicine, Shanghai 200030, China; licb@smhc.org.cn; 4 CAS Center for Excellence in Brain Science and Intelligence Technology (CEBSIT), Chinese Academy of Sciences, Shanghai 200031, China; 5 Institute of Psychology and Behavioral Science, Shanghai Jiao Tong University, Shanghai 200030, China; 6 Institute for Brain Research and Rehabilitation, South China Normal University, Guangzhou 510631, China; 7 Institutes of Psychological Sciences, Hangzhou Normal University, Hangzhou 311121, China; 8 Zhejiang Key Laboratory for Research in Assessment of Cognitive Impairments, Hangzhou 311121, China

**Keywords:** developmental dyslexia, prevalence, primary school children

## Abstract

Background: Developmental dyslexia (DD) is a specific learning disorder concerning reading acquisition that may has a lifelong negative impact on individuals. A reliable estimate of the prevalence of DD serves as the basis for diagnosis, intervention, and evidence-based health resource allocation and policy-making. Hence, the present meta-analysis aims to generate a reliable prevalence estimate of DD worldwide in primary school children and explore the potential variables related to that prevalence. Methods: Studies from the 1950s to June 2021 were collated using a combination of search terms related to DD and prevalence. Study quality was assessed using the STROBE guidelines according to the study design, with study heterogeneity assessed using the *I*^2^ statistic, and random-effects meta-analyses were conducted. Variations in the prevalence of DD in different subgroups were assessed via subgroup meta-analysis and meta-regression. Results: The pooled prevalence of DD was 7.10% (95% CI: 6.27–7.97%). The prevalence in boys was significantly higher than that in girls (boys: 9.22%, 95%CI, 8.07–10.44%; girls: 4.66%, 95% CI, 3.84–5.54%; *p* < 0.001), but no significant difference was found in the prevalence across different writing systems (alphabetic scripts: 7.26%, 95%CI, 5.94–8.71%; logographic scripts: 6.97%, 95%CI, 5.86–8.16%; *p* > 0.05) or across different orthographic depths (shallow: 7.13%, 95% CI, 5.23–9.30%; deep: 7.55%, 95% CI, 4.66–11.04%; *p* > 0.05). It is worth noting that most articles had small sample sizes with diverse operational definitions, making comparisons challenging. Conclusions: This study provides an estimation of worldwide DD prevalence in primary school children. The prevalence was higher in boys than in girls but was not significantly different across different writing systems.

## 1. Introduction

Developmental dyslexia (DD) is a specific impairment characterized by severe and persistent problems in the acquisition of reading skills; these problems are not caused by mental age, visual acuity problems, or inadequate schooling [1,2]. DD, also referred to as specific reading disability or specific reading disorder, is by far the most common type of learning disability, accounting for approximately 80% of all learning disabilities [3]. Due to their frustration with reading, a great number of dyslexic children are also at increased risk of academic and social problems [4]. These children often have higher reading anxiety [5,6,7], lower positive well-being [8], and experience negative attitudes [6,9].

Typically, children begin to be formally taught to read after entering primary school, and their word-reading ability reaches adult-like levels by the end of primary school [10]. Diagnosis of DD is normally achieved after a child begins structured schooling [11]. The primary school is, thus, an important point at which early literacy screening and interventions can help to identify potential reading difficulties and address risk factors [12,13]. Therefore, the present study focuses on DD in primary school children.

Dyslexia is fairly widespread but demonstrates uncertain prevalence, ranging from 5% to 17.5% [14,15], and the variability of prevalence may be related to several factors. First, different operational definitions may result in a different prevalence. The common sets of the cut-off for reading achievement are 1 and 1.5 standard deviations (SD) below the mean for the same age [16,17,18]. Second, environmental variables (e.g., regions, socioeconomic status) and other factors (e.g., grade, sub-deficit) may also influence each child’s risk of dyslexia.

Finally, it is particularly interesting to ask whether and in what way orthographic depth influences the prevalence of DD. On the one hand, logographic scripts may yield different prevalence estimates relative to alphabetic scripts. In alphabetic scripts in which the letters represent phonemes, the prevalence of DD was reported to range from 2.28% to 12.70% [19,20], even as high as 15% and 19.90% [21,22]. Unlike alphabetic scripts, logographic scripts such as Chinese have special language characteristics: (1) the smallest written units are characters representing monosyllabic morphemes; and (2) grapheme to phoneme mappings are created in an arbitrary way [23,24,25]. As logographic scripts, such as Chinese, require the memorization of picture-like characters by rote, it was previously believed that the script presented little or no difficulty in reading [26] until 1982, when Stevenson et al. [27] reported for the first time that DD did exist among Chinese and Japanese readers. On the other hand, even within alphabetic writing systems, such systems differ in terms of orthographic depths. According to the orthographic depth hypothesis (ODH) [28], shallower orthographies are easier to learn than deeper ones. For children, it is easier to learn how to map letters onto phonological forms that are known from speech in the shallower orthographies, where in units in the written language reliably correspond to units in the spoken language. In contrast, the other two theories (the psycholinguistic grain size theory and the grapholinguistic equilibrium hypothesis) propose that the incidence of DD will be very similar across both consistent and inconsistent orthographies but that its manifestation might differ according to orthographic consistency [29,30].

In addition, the gender ratio of DD is the subject of an ongoing debate [31,32,33]. Most studies reported that more boys suffered from DD than girls, and the gender ratio of boys to girls was about 3:1 [34,35,36], but some studies found no differences in the prevalence of DD between boys and girls [18,31]. The latter interpreted the over-representation of boys in DD prevalence to be a result of bias in behavioral observation [37]. To address this problem, we conducted a subgroup analysis of gender prevalence.

Taken together, a large number of previous studies have assessed the prevalence of DD in primary school children, but the results are largely mixed. More importantly, the previous review articles did not thoroughly discuss the prevalence of Chinese DD [14,15], although the number of Chinese users is large and widely distributed. Therefore, it is necessary to include Chinese for meta-analysis.

The present study thus aimed to conduct a systematic and meta-analytical review of previous studies that reported the prevalence of DD in children in primary school. More specifically, the present study aimed to address two issues: (a) what is the prevalence of childhood DD worldwide; and (b) whether the prevalence of DD varies according to gender, writing system, and other variables.

## 2. Materials and Methods

### 2.1. Search Strategy and Selection Criteria

This systematic review and meta-analysis was conducted in accordance with the preferred reporting items for systematic reviews and meta-analyses (PRISMA) reporting guidelines [38]. The protocol of this study was registered in PROSPERO (registration number: CRD42021232958).

Looking at studies from the 1950s to 10 June 2021, two researchers (X.L. and M.Z.) independently conducted a literature search of the China National Knowledge Infrastructure, Wanfang, CQ-VIP, the China Hospital Knowledge Database, EBSCO host, ProQuest, PubMed, Web of Science, the OATD database, Cochrane, Springerlink and EMBASE, using a combination of search terms related to DD (dyslexia, reading disability, reading disorder, or learning disability), and prevalence (prevalence, detectable rate, incidence rate, or epidemiology). Then, a search of the reference lists of the studies included in the first step was performed to complement our database searches. No language or time restrictions were applied. The full search strategies for different bibliographic databases are presented in Table A1.

The study inclusion criteria were that: (i) participants consisted of primary school students (age range: 6–13 years; grade range: 1st–6th); (ii) subjects were recruited through probability sampling methods; (iii) studies included DD prevalence as a main or secondary outcome; (iv) measures with good psychometrics were used to assess the symptoms of DD; (v) no restrictions in terms of languages and published periods. For studies involving both adolescents and primary school children, the data of the primary group had to be able to be disaggregated. For multiple articles that used data from the same investigation (duplicates), only the articles with the most comprehensive results or the largest sample size were kept.

The following studies were excluded: (i) those including non-primary school students as participants; (ii) case-control studies, randomized clinical trials, review articles, and editorials; (iii) gray literature-material published by governments, organizations, and industrial or commercial entities for non-academic purposes, conference proceedings, and abstracts; (iv) no reports on DD prevalence were included in the articles; (v) studies were of specific sub-populations of participants (e.g., participants with acute or chronic disease); (vi) the articles could not be retrieved in full-text form through online databases, via library requests or email correspondence with the authors of the studies; (vii) the articles provided insufficient data regarding sample information.

After removing duplicates from different bibliographic databases, the two researchers (X.L. and M.Z.) independently screened the titles and abstracts of all retrieved records from the literature search. Then, the same two researchers assessed the eligibility of potentially relevant articles in the full text against the selection criteria. A consensus was reached for any disagreements through discussion, or the matter was decided by the other two researchers (L.Y. and J.Z.).

### 2.2. Data Extraction and Quality Assessment

Data were independently extracted from the included articles by two researchers (Q.A. and Y.Z.). The collected information included title, first author, year of publication, country, study design, sampling strategy, diagnostic materials, diagnostic criteria, sample size, the number of participants screened as DD, and prevalence estimate. The regions of study location were designated as African Region, Region of the Americas, Southeast Asia Region, European Region, Eastern Mediterranean Region, and Western Pacific Region according to the World Health Organization (WHO) criteria and as high-income countries and low- and middle-income countries according to the World Bank (WB) criteria.

We rated the quality of included articles according to the Strengthening the Reporting of Observational Studies in Epidemiology (STROBE) reporting guideline in several dimensions: sample population, sample size, participation rate, outcome assessment, and analytical methods (Table A2) [39].

### 2.3. Overall Pooled Prevalence of DD

Before pooling the prevalence estimates, the variance of raw prevalence from each included study was stabilized, using the Freeman–Tukey double arc-sine transformation [40]. All estimates were presented after back transformation. We assessed the heterogeneity of prevalence estimates among studies using the Cochran Q test and *I*^2^ index [41,42]. For the Cochran Q test, *p* < 0.05 represented significant heterogeneity. For the *I*^2^ index, values of 25% or lower corresponded to low degrees of heterogeneity, 26% to 50%, to moderate degrees of heterogeneity, and values greater than 50% to high degrees of heterogeneity [41,42].

Because of high heterogeneity (as expected and observed), a random-effect meta-analysis (following the DerSimonian and Laird method) was used to calculate the overall pooled prevalence of DD with 95% CIs throughout this study [40]. To examine whether single studies had a disproportionally excessive influence, we applied a “leave-1-out” sensitivity analysis for each meta-analysis [43]. Publication bias in the meta-analysis was detected qualitatively by a visual inspection of funnel plots and quantitatively by the Egger linear regression test and the Begg rank correlation test when more than 10 estimates were available in a single analysis [44,45,46].

### 2.4. Subgroup Meta-Analysis and Meta-Regression of DD Prevalence

We conducted subgroup meta-analyses to determine potential sources of heterogeneity. As a rule, at least three studies should be available per subgroup.

Multiple data points were generally reported in a single study. To assess the associations among various sample characteristics and the prevalence of DD, we first conducted a univariable meta-regression, if possible, followed by a multi-variable meta-regression [47]. As a rule, at least 10 data points should be available for each variable in univariable meta-regression, and 20 in multivariable meta-regression [48,49]. Data were analyzed using RStudio, version 2021.09.1-372 (R Foundation for Statistical Computing).

## 3. Results

### 3.1. Study Selection and Characteristics

**Figure 1:**
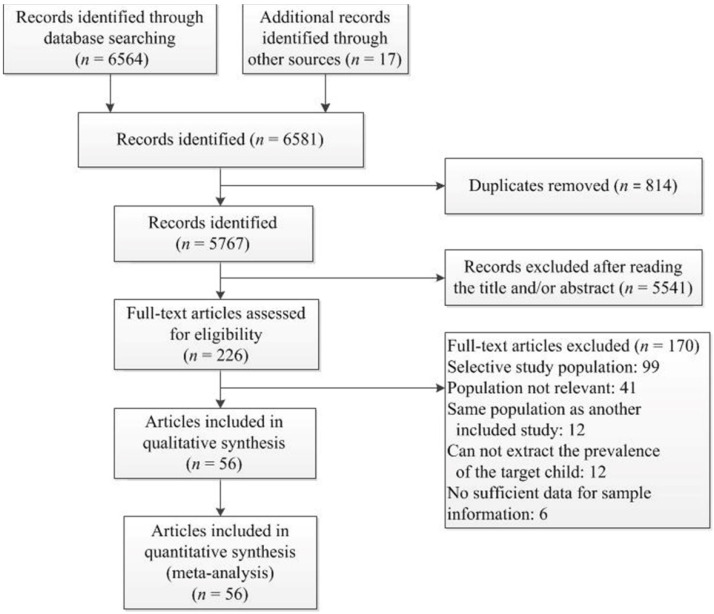
PRISMA flow diagram of literature search and study selection.

As outlined in Figure 1, our initial literature search identified a total of 6564 records. After applying the eligibility criteria, a final set of 56 articles, featuring 58 studies, were included in our quantitative synthesis. A list of the 56 included articles is given in Table A3.

The detailed characteristics of the included articles can be found in Table A3. In all, 41 of the 58 studies (70.69%) reported prevalence data for both boys and girls. Of the 58 studies, 27 (46.55%) were conducted among children using alphabetic scripts, while 31 (53.45%) were conducted among children using alphabetic scripts. In addition, grade 3 was the most-studied grade (21, 36.21%) and random sampling was the most-used method (37, 63.79%), while only four studies (6.90%) had a sample size greater than 10,000. Moreover, more than half of the 58 studies (33, 56.90%) were conducted in the Western Pacific area and in middle-income countries (40, 68.97%).

### 3.2. Pooled Prevalence of DD

**Figure 2:**
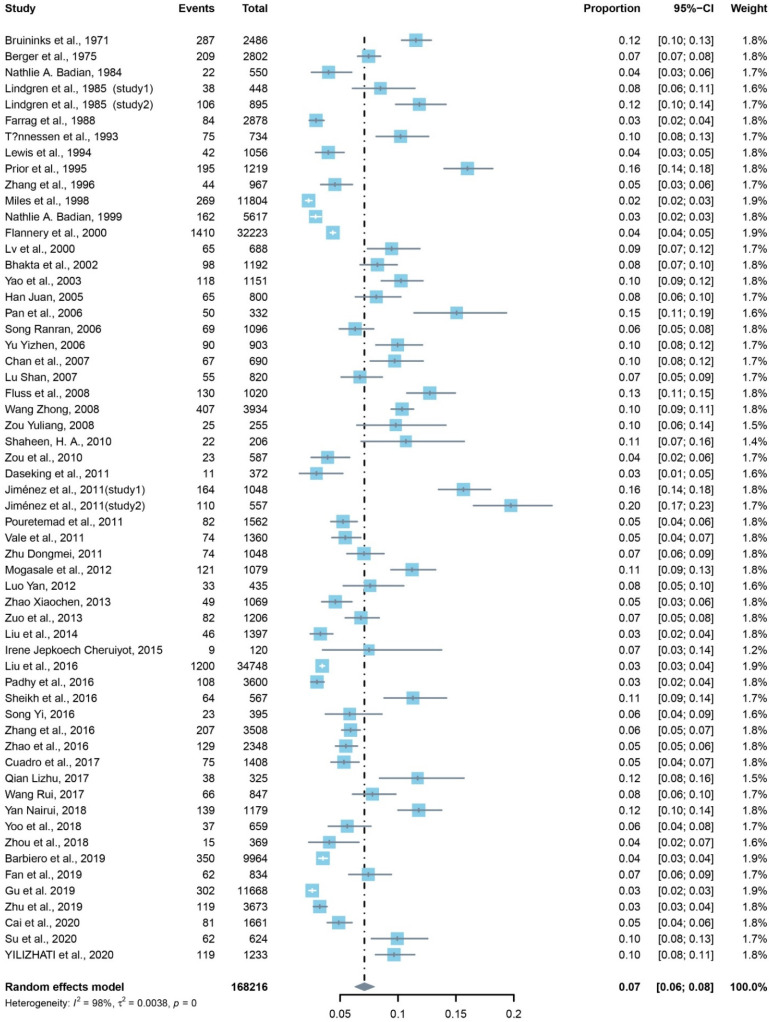
Forest plot for the prevalence of DD using random-effects meta-analysis.

Table 1 illustrates the results of overall and subgroup meta-analyses. Regarding DD, the pooled prevalence was 7.10% (95% CI: 6.27–7.97%), as ascertained using random-effects meta-analysis (Figure 2).

### 3.3. Sensitivity Analysis and Publication Bias

**Figure A1:**
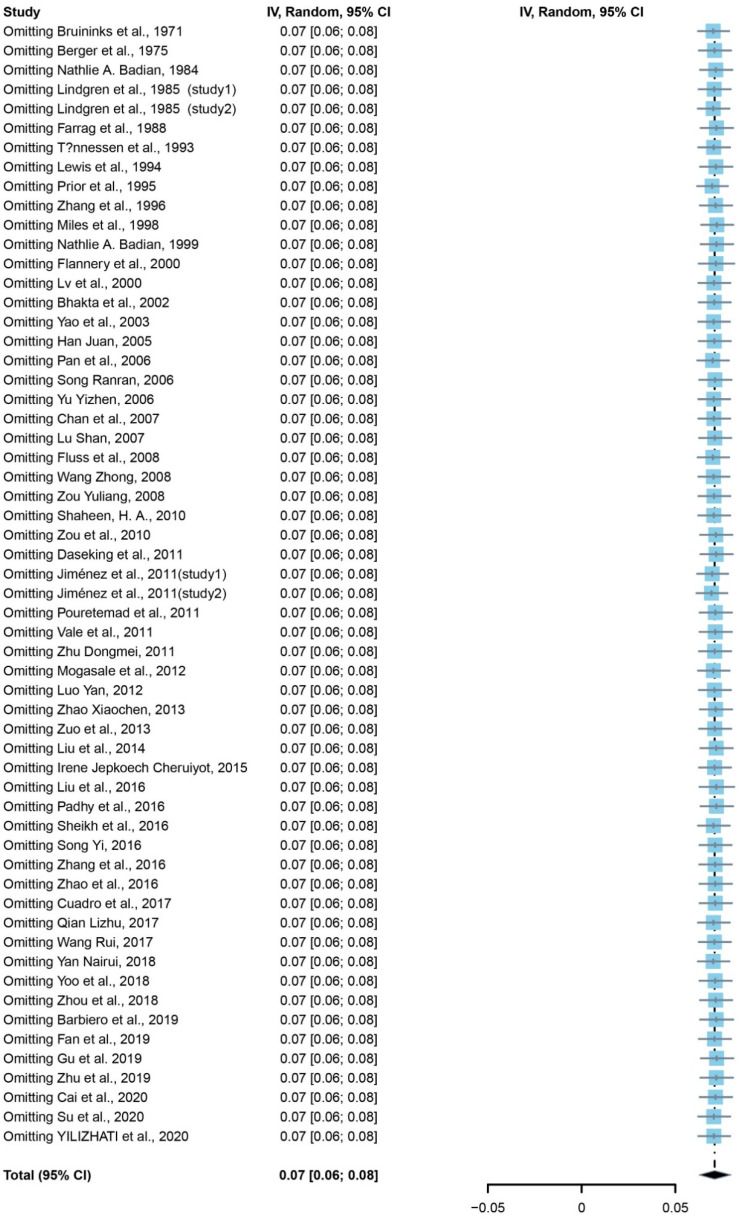
Leave-1-out forest.

**Figure A2:**
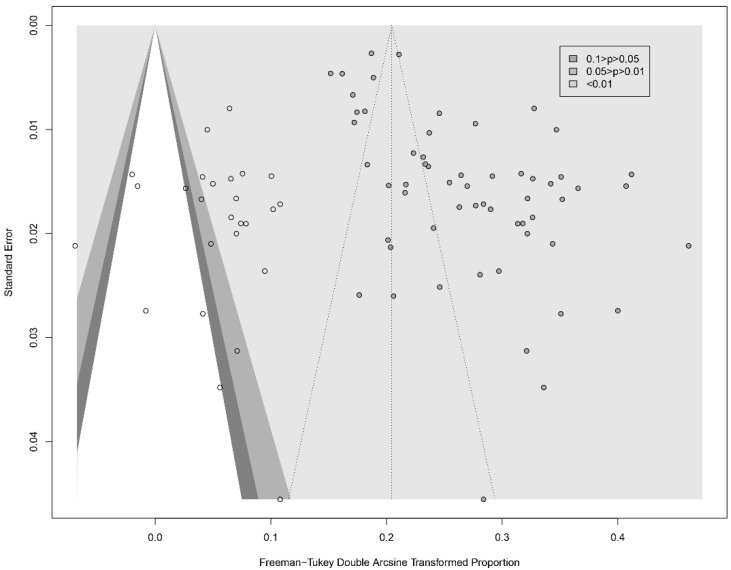
Funnel plot.

The “leave-1-out” sensitivity analysis showed that the pooled prevalence of DD varied from 6.93% (95% CI: 6.13–7.78%) to 7.21% (95% CI: 6.38–8.09%) after removing a single study at one time (Figure A1), indicating that no individual study significantly influenced the overall pooled prevalence in the meta-analysis. Publication bias was established based on the funnel plot (Figure A2), Egger test (*t* = 6.25, *p* < 0.001), and Begg test (*z* = 1.96, *p* = 0.05).

### 3.4. Subgroup Meta-Analysis and Meta-Regression of DD

**Figure 3:**
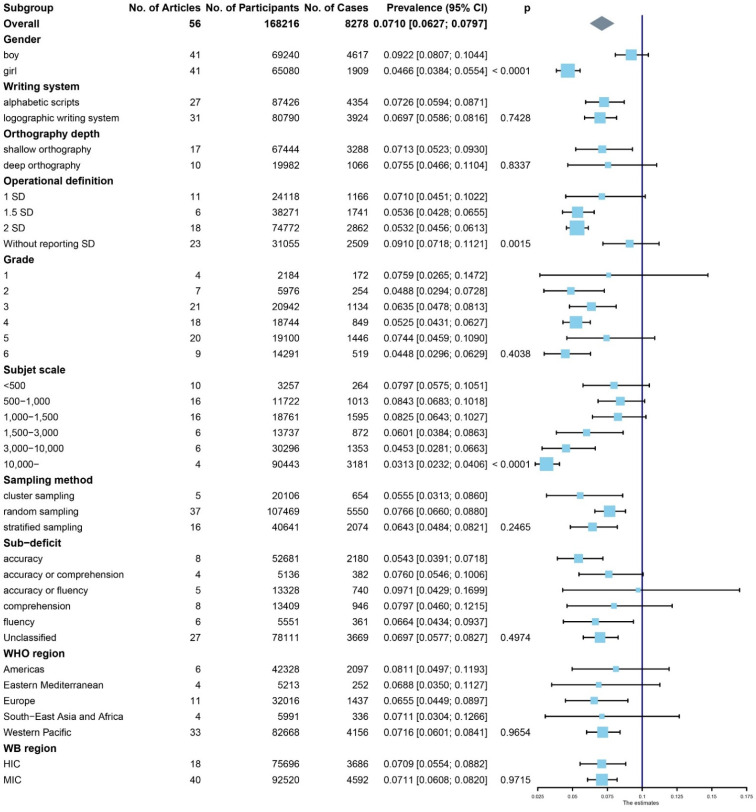
Forest plot for the subgroup meta-analysis of the prevalence of DD.

Table 1 and Figure 3 showed the prevalence of DD in different genders, writing systems, operational definitions, grades, sample sizes, sampling methods, sub-deficits, WHO regions, WB regions, and the forest plot for the difference in these factors.

There were significant differences in prevalence in terms of gender, operational definitions, and sample size. Specifically, the prevalence of DD was higher in boys (9.22%; 95% CI: 8.07–10.44%) than in girls (4.66%; 95% CI: 3.84–5.54%) (*p* < 0.001). In addition, a difference in DD prevalence was found among various operational definitions and sample sizes. The results of the post hoc analyses showed that DD prevalence was significantly lower when reporting 1.5 SD and 2SD as the cut-off values than without reporting the cut-off value (1.5 SD: 5.36%, 95% CI, 4.28–6.55%; 2 SD: 5.32%, 95% CI, 4.56–6.13%; without reporting SD: 9.10%, 95% CI, 7.18–11.21%; both *p* < 0.05, FDR-corrected). The prevalence in a large sample (more than 10,000) was significantly lower than that in smaller samples (500–1000 and 1000–1500) (10,000–: 3.13%, 95% CI, 2.32–4.06%; 500–1000: 8.43%, 95% CI, 6.83–10.18%; 1000–1500: 8.25%, 95% CI, 6.43–10.27%; both *p* = 0.09, FDR-corrected). However, there was no significant difference in the prevalence between the two smaller samples (*p* > 0.05). Univariate and multivariate regression results also showed that the subgroup of the largest sample size reported the lowest prevalence of DD.

Unexpectedly, the prevalence of DD did not differ significantly when it was stratified according to writing system (alphabetic scripts: 7.26%, 95% CI, 5.94–8.71%; logographic scripts: 6.97%, 95% CI, 5.86–8.16%; *p* > 0.05), or orthographic depth (shallow: 7.13%, 95% CI, 5.23–9.30%; deep: 7.55%, 95% CI, 4.66–11.04%; *p* > 0.05), or grade (grade 1: 7.59%, 95% CI, 2.65–14.72%; grade 2: 4.88%, 95% CI, 2.94–7.28%; grade 3: 6.35%, 95% CI, 4.78–8.13%; grade 4: 5.25%, 95% CI, 4.31–6.27%; grade 5: 7.44%, 95% CI, 4.59–10.90%; grade 6: 4.48%, 95% CI, 2.96–6.29%; *p* > 0.05). Similarly, there was no difference in the prevalence of DD among different subgroups of sub-deficits, sampling methods, WHO regions, and WB regions (*p* > 0.05).

## 4. Discussion

This systematic review and meta-analysis estimated the worldwide prevalence of DD in primary school children, with a prevalence of 7.10% (95% CI: 6.27–7.97%). There was a significant gender difference, and the gender ratio of boys to girls was about 2:1. However, there was no language-specific difference in the prevalence of DD. In addition, the prevalence was influenced by operational definition and sample size, but not by sub-deficits, grade, sampling method, WHO region or WB region. To our best knowledge, this is the first synthesized analysis on the prevalence of DD.

The pooled prevalence of 7.10% (95% CI: 6.27–7.97%) that is estimated in the present study is within the range of previous selective reviews, which have suggested that the prevalence of DD was in the range of 5–17.5% [14,15]. This is likely due to the similar diagnostic criteria of DD in most of the previous studies, in which DD was mainly defined as the low end of a normal distribution of word-reading ability [50]. Many disorders do not represent categories but instead the extremes on a continuous distribution that ranges from optimal outcomes to poor outcomes, with the underlying causal mechanisms being similar across the whole distribution. Essentially, most behaviorally defined disorders, including DD, are continuous disorders. In the present study, we were able to pool the prevalence of DD in children based on the available evidence, which allowed our systematic review and meta-analysis to provide a more comprehensive estimate of the prevalence of DD.

Interestingly, our calculation of the gender ratio regarding DD of boys to girls is about 2:1 (boys: 9.22%; 95% CI: 8.07–10.44%; girls: 4.66%; 95% CI: 3.84–5.54%) (*p* < 0.001). This result is consistent with previous studies that reported a higher prevalence of DD for boys than for girls [31,35,51]. One explanation for this gender difference in DD prevalence is that some teachers are more likely to refer boys for assessment as having special problems because boys are often perceived as being more disruptive than girls [52]. However, focusing on large-scale epidemiological studies that were not based on school-referred samples, Rutter and his colleagues (2007) also found that boys were more likely than girls to have a reading disability, indicating that teacher bias cannot account entirely for gender difference [53]. A similar phenomenon is also found in logographic writing systems [54,55]. Other explanations come from biological and environmental hypotheses, including genetic causes [56,57], immunological factors, perinatal complications, differences in brain functioning due to differential exposure or sensitivity to androgens [58], and differential resilience to neural insult [59]. Our current study cannot provide enough evidence to support or reject any of the above hypotheses; therefore, more studies on DD in both boys and girls are needed in the future. At the same time, the current findings suggest that teachers may need to pay more attention to boys who exhibit reading difficulties or disorders.

Another important finding is that the prevalence of DD did not differ significantly when stratified by writing system (alphabetic scripts: 7.26%, 95% CI, 5.94–8.71%; logographic scripts: 6.97%, 95% CI: 5.86–8.16%; *p* = 0.74). This is an unexpected result since logographic scripts are very distinctive (such as arbitrary mapping between the graphic and sound forms of words) relative to alphabetic scripts from the perspective of language; therefore, some experts believe that DD may be absent or rare in logographic scripts [26]. Research on DD has been initially and mainly conducted among the users of alphabetic scripts. Until the 1980s, researchers examined large samples of fifth-grade children in Japan, Taiwan, and the United States using a reading test and a battery of 10 cognitive tasks. However, the results showed that the prevalence of DD in Japan, Taiwan, and the United States was 5.4%, 7.5%, and 6.3%, respectively, suggesting that there is no significant difference in the prevalence of DD among different writing systems [27]. One explanation for this and our current findings is that the similarity in DD prevalence across different writing systems may be related to cross-cultural universality in the neurobiological and neurocognitive underpinnings of DD [15]. Some Western researchers and writers believed that Chinese characters are derived from pictographs, but this is not true. Instead, Chinese orthography is not primarily pictographic [27].

In addition, we found that DD prevalence did not differ across languages with different orthographic depths (shallow: 7.13%, 95% CI, 5.23–9.30%; deep: 7.55%, 95% CI, 4.66–11.04%; *p* > 0.05). These findings support the psycholinguistic grain size theory rather than the orthographic depth hypothesis [28,29]. When the orthography of the language is relatively shallow, readers can focus exclusively on the small psycholinguistic grain size of the phoneme. Otherwise, they will learn additional correspondences for larger orthographic units, such as syllables, rhymes, or whole words. Therefore, the prevalence of DD is very similar in both consistent and inconsistent orthographies, but its manifestations may vary according to orthographic depth.

Remarkably, operational definitions significantly affected the prevalence of DD. The present study found that studies with stricter operational definitions reported lower prevalence. Specifically, DD prevalence was significantly lower when using 1.5 SD and 2SD as the cut-off values than when not reporting SD (1.5 SD: 5.36%, 95% CI, 4.28–6.55%; 2 SD: 5.32%, 95% CI, 4.56–6.13%; without reporting SD: 9.10%, 95% CI, 7.18–11.21%; both *p* < 0.05, FDR-corrected). This finding is consistent with a recent selective review, suggesting that the prevalence depends on the severity of the reading problem—with lower rates for more severe problems [16]. Although the recognition of DD dates back over a century, no consensus has been reached regarding its diagnostic criteria. Therefore, many studies even use scores below 20% [60], scores in the bottom 10% [61], using different materials, and many other cut-offs for convenience. Essentially, all behaviorally defined disorders, including DD, are continuous disorders, and their operational definitions are found to be confusing in the current study. Perhaps now is not the time for change, with the continuous development of theoretical and empirical research; perhaps there will be a more appropriate operational definition for DD in the future.

It is worth noting that studies with more than 10,000 subjects reported a lower average prevalence of DD when compared to studies with 500–1000 and 1000–1500 subjects. By reviewing these studies, we found that the large sample-size studies have a common feature: that is, the diagnostic criteria were relatively strict. Only students who scored 1.5 or even 2 SD below the average on diagnostic tests were diagnosed as having DD [35,62,63]. Because of their strict diagnostic criteria, the prevalence was significantly lower than that of other subgroups [18,20]. Interestingly, in studies on other disorders, such as Tourette’s syndrome, epidemiological investigations also demonstrated that studies with larger sample sizes tended to report a relatively lower prevalence [64,65], although the reason is not clear.

There was no grade difference in DD prevalence. In the literature, the association between grade and DD prevalence remains unclear. Some studies reported that DD prevalence was lower in higher grades than in lower grades [66], and explained this finding with the argument that DD symptoms improve through systematic learning [14]. Several studies, however, have shown a higher DD prevalence in higher grades, relative to that observed in lower grades [67]. In addition, most studies reported no difference in DD prevalence among different grades [68,69,70]. Studies have shown that the level of reading ability in the first few years of school will continue in the following years and that the DD prevalence during schooling does not change greatly [20,37]. Most previous studies only studied the prevalence of DD in specific grades, mainly in grades 3 to 5, which makes it difficult to directly and empirically address the above issue [55,70,71]. In order to examine whether and how DD prevalence changes with progression through grades, future studies need to include all grades of elementary school and make the sample sufficiently representative. There was also no difference in the prevalence of sub-deficits. This shows that different tests and different indicators have no effect on the prevalence rate. That is, when there is a problem with accuracy, there is usually a problem with fluency or comprehension, and dyslexia shows no obvious differentiation.

**Figure A3:**
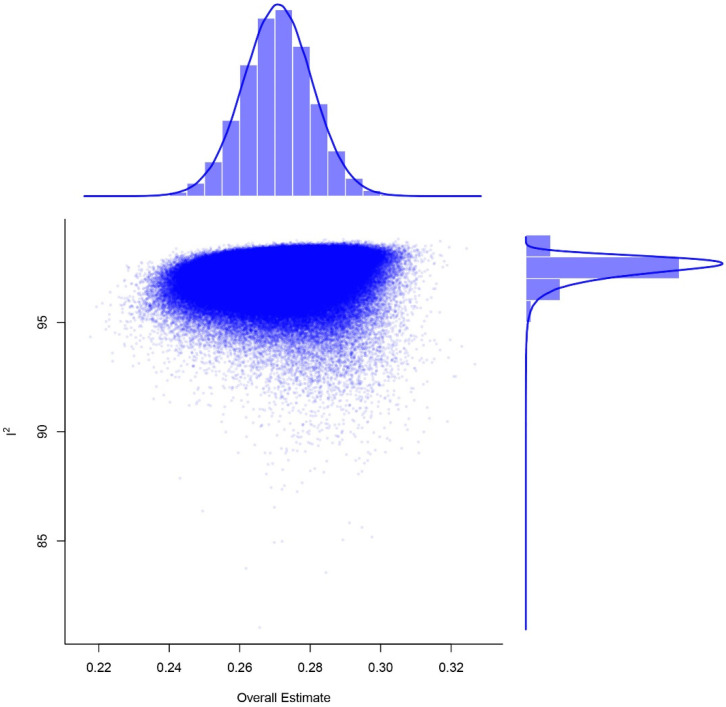
GOSH plot.

As expected, we found significant heterogeneity when pooling the prevalence rates of DD. Thus, we performed sensitivity analyses, subgroup analyses, and meta-regression on many variables. After omitting each study one at a time (leave-1-out forest), the pooled prevalence of DD was shown to be robust and consistent. That is, no one study in this meta-analysis exerted a very high influence on our overall results. Under this condition, we further explored the patterns of effect sizes and heterogeneity in our data through a graphic display of heterogeneity (GOSH) plots [72] and found that all included studies had a low effect size and high heterogeneity (Figure A3). This result was consistent with the results of subgroup analysis, i.e., each subgroup had high heterogeneity (Table 1). In meta-regression, only the *p*-value of the sample size reached a significant level, which could explain the 39.56% heterogeneity (R^2^ = 39.56%). This indicates that the large variations in sample size among different studies may be an important reason for their heterogeneity. Another reason for heterogeneity may be that children were drawn from studies performed in a wide variety of countries with differing cultural, ethnic, social, and economic characteristics. In conclusion, such high heterogeneity in epidemiological meta-analysis is not unexpected. However, the results of this study should be interpreted with caution.

The strengths of this study include the comprehensive search strategies, a double review process, and stringent selection criteria. In our systematic review, we included only studies that were conducted in standard primary schools so that the generalizability of our results could be fully guaranteed. Moreover, we were able to pool the prevalence of DD in the included children based on the available evidence, which allowed our systematic review and meta-analysis to cover a broad scope regarding the prevalence of childhood DD.

Several intrinsic limitations of this study should also be acknowledged. First, the pooled prevalence of DD in the studied children might be affected by publication bias. We tried to minimize publication bias by searching for non-English literature and conference abstracts. Unfortunately, we could not completely rule out publication bias because of the observational nature of our study. Second, there were inherent disadvantages in pooling prevalence reports from disparate studies. For DD, sufficient data were available to pool the prevalence estimates. However, our subgroup analysis on the prevalence of any DD according to grade group, region group, and income group were only based on a limited number of studies that provided corresponding prevalence numbers. Third, ten variables across the included studies were systematically assessed, and only those studies with a large sample size were identified as showing a lower prevalence of DD. Previous studies [73,74] have suggested that socioeconomic factors were likely to contribute to disparities in DD prevalence rates in different subgroups. However, only high- and middle-income countries were assessed in the current study. Future studies are needed to explain the heterogeneity. More high-quality epidemiologic investigations on DD appear to be necessary, especially regarding different grades and in low-income countries.

## 5. Conclusions

This systematic review and meta-analysis is the first study to estimate the worldwide prevalence of DD. The results suggested that DD represents a considerable public health challenge worldwide (with a prevalence of 7.10%, 95% CI: 6.27–7.97%) and boys seem to be more affected than girls. There was no significant difference in the prevalence of DD either between logographic and alphabetic writing systems or between alphabetic scripts with different orthographic depths. However, a clear operational definition is urgently needed for the diagnosis of DD.

## Figures and Tables

**Table 1:** Prevalence of DD using random-effects meta-analysis and subgroup meta-analysis.

Variable	No. of Studies	Prevalence (95% CI)	*I*^2^, %	*p*-Value			
				Q Test	Egger Test	Begg Test	Subgroup Difference
Global Analysis for DD							
DD	56	7.10 [6.27; 7.97]	97.60	<0.001	<0.001	0.05	NA
Gender							
boy	41	9.22 [8.07; 10.44]	95.80	<0.001	<0.001	0.35	<0.001
girl	41	4.66 [3.84; 5.54]	95.20	<0.001	<0.001	0.17	
Writing system							
alphabetic scripts	27	7.26 [5.94; 8.71]	98.10	<0.001	<0.05	0.06	0.74
logographic writing system	31	6.97 [5.86; 8.16]	96.90	<0.001	<0.001	0.27	
Orthography depth							
shallow orthography	17	7.13 [5.23; 9.30]	98.30	<0.001	<0.05	0.19	0.83
deep orthography	10	7.55 [4.66; 11.04]	97.80	<0.001	<0.05	0.24	
Operational definition							
1 SD	11	7.10 [4.51; 10.22]	98.40	<0.001	<0.01	0.14	<0.01
1.5 SD	6	5.36 [4.28; 6.55]	87.70	<0.001	NA	NA	
2 SD	18	5.32 [4.56; 6.13]	93.70	<0.001	<0.01	0.18	
Without reporting SD	23	9.10 [7.18; 11.21]	97.20	<0.001	0.03	0.58	
Grade							
1	4	7.59 [2.65; 14.72]	96.40	<0.001	NA	NA	0.40
2	7	4.88 [2.94; 7.28]	92.00	<0.001	NA	NA	
3	21	6.35 [4.78; 8.13]	95.20	<0.001	0.06	0.15	
4	18	5.25 [4.31; 6.27]	85.00	<0.001	0.03	0.12	
5	20	7.44 [4.59; 10.90]	98.20	<0.001	0.47	0.01	
6	9	4.48 [2.96; 6.29]	93.20	<0.001	NA	NA	
Sample size							<0.001
<500	10	7.97 [5.75; 10.51]	84.00	<0.001	0.50	0.53	
500–1000	16	8.43 [6.83; 10.18]	90.90	<0.001	0.59	0.72	
1000–1500	16	8.25 [6.43; 10.27]	95.80	<0.001	0.15	0.22	
1500–3000	6	6.01 [3.84; 8.63]	97.20	<0.001	NA	NA	
3000–10,000	6	4.53 [2.81; 6.63]	98.40	<0.001	NA	NA	
10,000–	4	3.13 [2.32; 4.06]	98.10	<0.001	NA	NA	
Sampling method							
cluster sampling	5	5.55 [3.13; 8.60]	98.10	<0.001	NA	NA	0.25
random sampling	37	7.66 [6.60; 8.80]	97.20	<0.001	<0.001	0.80	
stratified sampling	16	6.43 [4.84; 8.21]	97.80	<0.001	<0.05	0.05	
Sub-deficits							
accuracy	8	5.43 [3.91; 7.18]	97.80	<0.001	NA	NA	0.50
accuracy or comprehension	4	7.60 [5.46; 10.06]	88.00	<0.001	NA	NA	
accuracy or fluency	5	9.71 [4.29; 16.99]	98.80	<0.001	NA	NA	
comprehension	8	7.97 [4.60; 12.15]	98.30	<0.001	NA	NA	
fluency	6	6.64 [4.34; 9.37]	92.40	<0.001	NA	NA	
Unclassified	27	6.97 [5.77; 8.27]	97.30	<0.001	<0.001	0.44	
WHO region							
Americas	6	8.11 [4.97; 11.93]	98.80	<0.001	NA	NA	0.97
Eastern Mediterranean	4	6.88 [3.50; 11.27]	95.90	<0.001	NA	NA	
Europe	11	6.55 [4.49; 8.97]	98.20	<0.001	<0.05	0.31	
South-East Asia and Africa	4	7.11 [3.04; 12.66]	97.50	<0.001	NA	NA	
Western Pacific	33	7.16 [6.01; 8.41]	97.30	<0.001	<0.001	0.44	
WB region							
HIC	18	7.09 [5.54; 8.82]	98.40	<0.001	<0.01	0.43	0.97
MIC	40	7.11 [6.08; 8.20]	97.00	<0.001	<0.001	0.07	

Abbreviations: WHO, World Health Organization; WB, World Bank; HIC, high-income countries; MIC, middle-income countries; NA, not applicable.

## Data Availability

All data related to the research are presented in the article.

## Appendix A

**Table A1:** Search strategy.

Database	Search Strategy
China National Knowledge Infrastructure	TI = ‘阅读障碍’ + ’发展性阅读障碍’ + ’特异性阅读障碍’ + ’词盲’ + ’阅读困难’ + ’学习障碍’ AND AB = ‘流行病学’ + ’患病率’ + ’检出率’ + ’发生率’ + ’发病率’ (TI = ‘Dyslexia’ + ‘reading disabilit*’ + ‘reading disorder*’ + ‘word blindness’ + ‘specific reading retardation’ + ‘backward reading’ + ‘reading difficult*’ + ‘learning disabilit*’ AND AB = ‘prevalence’ + ‘detectable rate’ + ‘incidence rate’ + ‘epidemiology’)
Wanfang	题名:(“阅读障碍” or “发展性阅读障碍” or “特异性阅读障碍” or “词盲” or “阅读困难” or “学习障碍”) and 摘要:(“患病率” or “检出率” or “发病率” or “流行病学” or “发生率”) [title: (“Dyslexia” or “reading disabilit*” or “reading disorder*” or “word blindness” or “specific reading retardation” or “backward reading” or “reading difficult*” or “learning disabilit*”) and abstract: (“prevalence” or “detectable rate” or “incidence rate” or “epidemiology”)]
CQ-VIP	(R = 阅读障碍 + R = 发展性阅读障碍 + R = 特异性阅读障碍 + R = 词盲 + R = 阅读困难 + R = 学习障碍) AND (U = 患病率 + U = 检出率 + U = 发病率 + U = 流行病学 + U = 发生率) [(R = Dyslexia + R = reading disabilit* + R = reading disorder* + R = word blindness + R = specific reading retardation + R = backward reading + R = reading difficult* + R = learning disabilit*) AND (U = prevalence + U = detectable rate + U = incidence rate + U = epidemiology)]
China Hospital Knowledge Database	TI = ‘阅读障碍’ + ’发展性阅读障碍’ + ’特异性阅读障碍’ + ’词盲’ + ’阅读困难’ + ’学习障碍’ AND TI = ‘流行病学’ + ’患病率’ + ’检出率’ + ’发生率’ + ’发病率’ (TI = ‘Dyslexia’ + ‘reading disabilit*’ + ‘reading disorder*’ + ‘word blindness’ + ‘specific reading retardation’ + ‘backward reading’ + ‘reading difficult*’ + ‘learning disabilit*’ AND TI = ‘prevalence’ + ‘detectable rate’ + ‘incidence rate’ + ‘epidemiology’)
EBSCO Host	TI ((Dyslexia OR (reading disabilit*) OR (reading disorder*) OR (word blindness) OR (specific reading retardation) OR (backward reading) OR (reading difficult*) OR (learning disabilit*)) AND AB ((prevalence OR (detectable rate) OR (incidence rate) OR epidemiology))
Proquest	((dyslexia) [SU] OR (reading disabilit*) [SU] OR (reading disorder*) [SU] OR (word blindness) [SU] OR (specific reading retardation) [SU] OR (backward reading) [SU] OR (reading difficult*) [SU] OR (learning disabilit*) [SU]) AND ((prevalence) [FT°] OR (detectable rate) [FT°] OR (incidence rate) [FT°] OR (epidemiology) [FT°])
PubMed	(“dyslexia” [Title] OR “reading disabilit*” [Title] OR “reading disorder*” [Title] OR “word blindness” [Title] OR “specific reading retardation” [Title] OR “backward reading” [Title] OR “reading difficult*” [Title] OR “learning disabilit*” [Title]) AND (“prevalence” [Title/Abstract] OR “detectable rate” [Title/Abstract] OR “incidence rate” [Title/Abstract] OR “epidemiology” [Title/Abstract])
Web of Science	TI = (Dyslexia OR (reading disabilit*) OR (reading disorder*) OR (word blindness) OR (specific reading retardation) OR (backward reading) OR (reading difficult*) OR (learning disabilit*)) AND AB = (prevalence OR (detectable rate) OR (incidence rate) OR epidemiology)
OATD database	abstract:(dyslexia OR “reading disabilit*” OR “reading disorder*” OR “word blindness” OR “specific reading retardation” OR “backward reading” OR “reading difficult*” OR “learning disabilit*” OR “reading difficult*”) AND (prevalence OR “detectable rate” OR “incidence rate” OR epidemiology)
Cochrane	(‘dyslexia’ OR ‘reading disabilit*’ OR ‘reading disorder*’ OR ‘word blindness’ OR ‘specific reading retardation’ OR ‘backward reading’ OR ‘reading difficult*’ OR ‘learning disabilit*’) in Title Abstract Keyword AND (‘prevalence’ OR ‘detectable rate’ OR ‘incidence rate’ OR ‘epidemiology’) in Abstract
Springerlink	TI(“dyslexia” OR “reading disabilit*” OR “reading disorder*” OR “word blindness” OR “specific reading retardation” OR “backward reading” OR “reading difficult*” OR “learning disabilit*”) AND AB(“prevalence” OR “detectable rate” OR “incidence rate” OR “epidemiology”)
EMBASE	((dyslexia OR ‘reading disabilit*’ OR ‘reading disorder*’ OR ‘word blindness’ OR ‘specific reading retardation’ OR ‘backward reading’ OR ‘reading difficult*’ OR ‘learning disabilit*’):ti) AND ((prevalence OR ‘detectable rate’ OR ‘incidence rate’ OR epidemiology):ab)

“*” was used to replace zero, single or multiple characters.

**Table A2:** Quality scores.

ID	Author	Year Published	Quality Score					
			Sample Population	Sample Size	Participation	Outcome Assessment	Analytical Methods	Total Score
1	Bruininks et al., 1971	1971	2	1	2	2	2	9
2	Berger et al., 1975	1975	2	1	2	2	2	9
3	Nathlie A. Badian, 1984	1984	1	0	2	2	2	7
4	Lindgren et al., 1985	1985	2	1	1	2	2	8
5	Farrag et al., 1988	1988	2	1	2	2	2	9
6	Tonnessen et al., 1993	1993	2	1	2	2	2	9
7	Lewis et al., 1994	1994	2	1	2	2	2	9
8	Prior et al., 1995	1995	2	1	2	2	2	9
9	Zhang et al., 1996	1996	2	1	2	2	2	9
10	Miles et al., 1998	1998	2	1	2	2	2	9
11	Nathlie A. Badian, 1999	1999	1	1	2	2	2	8
12	Lv et al., 2000	2000	1	0	2	1	2	6
13	Flannery et al., 2000	2000	2	1	2	2	2	9
14	Bhakta et al., 2002	2002	2	1	1	2	2	8
15	Yao et al., 2003	2003	2	0	2	1	2	7
16	Han Juan, 2005	2005	1	0	2	2	2	7
17	Pan et al., 2006	2006	1	0	2	1	2	6
18	Song Ranran, 2006	2006	2	0	2	1	2	7
19	Yu Yizhen, 2006	2006	1	0	2	1	2	6
20	Chan et al., 2007	2007	2	0	2	2	2	8
21	Lu Shan, 2007	2007	2	0	2	1	2	7
22	Fluss et al., 2008	2008	2	2	2	2	2	10
23	Wang Zhong, 2008	2008	2	0	2	1	2	7
24	Zou Yuliang, 2008	2008	2	0	2	1	2	7
25	Shaheen, H.A., 2010	2010	1	0	2	2	1	6
26	Zou et al., 2010	2010	1	0	2	1	2	6
27	Daseking et al., 2011	2011	1	0	2	2	1	6
28	Jiménez et al., 2011	2011	2	1	2	2	2	9
29	Pouretemad et al., 2011	2011	2	0	2	2	2	8
30	Vale et al., 2011	2011	2	1	2	2	2	9
31	Zhu Dongmei, 2011	2011	2	0	2	2	2	8
32	Mogasale et al., 2012	2011	2	1	2	2	2	9
33	Luo Yan, 2012	2012	1	0	2	1	2	6
34	Zhao Xiaochen, 2013	2013	1	0	2	2	2	7
35	Zuo et al., 2013	2013	1	0	2	1	2	6
36	Liu et al., 2014	2014	1	0	2	2	2	7
37	Irene Jepkoech Cheruiyot, 2015	2015	1	1	2	2	2	8
38	Liu et al., 2016	2016	1	0	2	1	2	6
39	Padhy et al., 2016	2016	2	2	2	1	1	8
40	Sheikh et al., 2016	2016	2	1	2	2	2	9
41	Song Yi, 2016	2016	2	0	2	1	2	7
42	Zhang et al., 2016	2016	2	0	2	1	2	7
43	Zhao et al., 2016	2016	1	0	2	1	2	6
44	Cuadro et al., 2017	2017	1	0	2	2	2	7
45	Qian Lizhu, 2017	2017	1	0	2	1	2	6
46	Wang Rui, 2017	2017	1	0	2	2	1	6
47	Yan Nairui, 2018	2018	1	0	2	1	2	6
48	Yoo et al., 2018	2018	1	0	2	2	2	7
49	Zhou et al., 2018	2018	1	0	2	1	2	6
50	Barbiero et al., 2019	2019	1	2	2	2	1	8
51	Fan et al., 2019	2019	1	0	2	2	1	6
52	Gu et al., 2019	2019	1	0	2	1	2	6
53	Zhu et al., 2019	2019	1	0	2	1	2	6
54	Cai et al., 2020	2020	1	2	2	2	2	9
55	Su et al., 2020	2020	1	0	2	1	2	6
56	Yilizhati Maimaiti et al. 2020	2020	1	0	2	2	2	7

**Table A3:** Characteristics of included articles.

ID	Author (Year)	Country	Sampling Strategy	Writng System	Ozone (WHO)	Income (WB)	Diagnostic Materials	Diagnostic Criteria	Sample Size	Prevalence Number	Prevalence Rate
1	Bruininks et al., 1971	USA	random sampling	alphabetic script	Americas	HIC	(1) The Lorge-Thorndike intelligence tests; (2) the reading comprehension and arithmetic computation subtest of the Iowa Tests of Basic Skills	(1) IQ ≥ 80; (2) one grade or more below the expected achievement in a reading test	Total = 2486 boys = 1233 girls = 1253 3rd = 1303 6th = 1183	Total = 287 boys = 186 girls = 101 3rd = 202 6th = 85	Total = 11.54% boys = 15.09% girls = 8.06% 3rd = 15.50% 6th = 7.19%
2	Berger et al., 1975	Great Britain	random sampling	alphabetic script	Europe	HIC	(1) The NFER test NV5; (2) the Watts-Vernon test SRI; (3) the NFER test SRA; (4) the short form of the WISC; (5) the Neale Analysis of Reading Ability	(1) SRA ≤ 15 or SRI ≤ 10; (2) scores on either the accuracy or comprehension scales on the Neale Test fell 30 months or more below those predicted	Total = 2802 boys = 1428 girls = 1374	Total = 209 boys = 156 girls = 53	Total = 7.46% boys = 10.92% girls = 3.86%
3	Nathlie A. Badian, 1984	USA	random sampling	alphabetic script	Americas	HIC	(1) The Stanford achievement test, SAT; (2) the Wechsler intelligence scale for children–revised, WISC-R	(1) Total reading score ≤ 20 percentile on SAT; (2) IQ ≥ 85	Total = 550 boys = 284 girls = 266	Total = 22 boys = 16 girls = 6	Total = 4.00% boys = 5.63% girls = 2.26%
4	Lindgren et al., 1985 (study1)	USA	cluster sampling	alphabetic script	Americas	HIC	(1) The IEA reading test; (2) the short form of the Wechsler intelligence scale for children	Reading score < 85 and either VIQ or PIQ ≥ 90	Total = 895	Total = 106	Total = 11.84%
4	Lindgren et al., 1985 (study2)	Italy	stratified sampling	alphabetic script	Europe	HIC	(1) The IEA reading test; (2) the short form of the Wechsler intelligence scale for children	Reading score < 85 and either VIQ or PIQ ≥ 90	Total = 448	Total = 38	Total = 8.48%
5	Farrag et al., 1988	Egypt	stratified sampling	alphabetic script	Eastern Mediterranean	MIC	(1) The modified Alaska letters identification test (ALIT); (2) the Assiut dyslexia screening test (ADST); (3) the Stanford–Binet IQ test	Reading scores of less than 142 and IQ levels of 90 or more.	Total = 2878 boys = 1610 girls = 1268	Total = 84 boys = 57 girls = 27	Total = 2.92% boys = 3.54% girls = 2.13%
6	Tønnessen et al., 1993	Norway	cluster sampling	alphabetic script	Europe	HIC	(1) The silent word recognition test; (2) the phonological decoding test	Scored below 20% on two tests	Total = 734 boys = 394 girls = 340	Total = 75 boys = 50 girls = 25	Total = 10.22% boys = 12.69% girls = 7.35%
7	Lewis et al., 1994	Great Britain	cluster sampling	alphabetic script	Europe	HIC	(1) Young’s (1970) group mathematics test (GMT); (2) Young’s (1976) SPAR (spelling and reading) test; (3) Raven’s colored progressive matrices (CPM)	Scored above 90 on arithmetic and nonverbal intelligence tests, but scored below 85 on reading, have no sensory or perceptual handicap, no psychiatric disturbance history, and English is the first language	Total = 1056 boys = 559 girls = 497	Total = 42 boys = 32 girls = 10	Total = 3.98% boys = 5.72% girls = 2.01%
8	Prior et al., 1995	Australia	random sampling	alphabetic script	Western Pacific	HIC	(1) ACER word knowledge test; (2) Rurrer child behavior scales A and B	Scored more than 1 SD below the grade-2 mean on the reading test	Total = 1219	Total = 195	Total = 16.00%
9	Zhang et al., 1996	China	stratified sampling	logographic script	Western Pacific	MIC	(1) A self-compiled reading achievement inventory; (2) combined Raven’s test (city edition)	Children’s reading achievement was more than 2SD below the average for their grade	Total = 967	Total = 44	Total = 4.55%
10	Miles et al., 1998	Great Britain	cluster sampling	alphabetic script	Europe	HIC	(1) The shortened Edinburgh reading test; (2) the Bangor dyslexia test (left–right, months forward, and months reversed); (3) the recall of digits subtest from the British ability scales (BAS)	(1) On the word recognition test, outliers beyond 1.5 standard deviations from the mean were excluded; (2) those children whose residuals were ≥ 1.0 SD were described as “underachievers”	Total = 11,804 boys = 5995 girls = 5809	Total = 269 boys = 223 girls = 46	Total = 2.28% boys = 3.72% girls = 0.79%
11	Nathlie A. Badian, 1999	USA	cluster sampling	alphabetic script	Americas	HIC	(1) The Wechsler preschool and primary scale of intelligence (WPPSI); (2) the Stanford achievement test (SAT); (3) the Wechsler intelligence scale for children–revised (WISC-R)	(1) A reading comprehension score of less than the 25th percentile (< 90) on the SAT; (2) scores were 1.5 SDs or more below the expected level, based on listening comprehension	Total = 5617 1st = 903 2nd = 919 3rd = 988 4th = 896 5th = 908 6th = 1003	Total = 162 1st = 28 2nd = 27 3rd = 28 4th = 33 5th = 32 6th = 14	Total = 2.88% 1st = 3.10% 2nd = 2.94% 3rd = 2.83% 4th = 3.68% 5th = 3.52% 6th = 1.40%
12	Flannery et al., 2000	USA	random sampling	alphabetic script	Americas	HIC	(1) The Weschler intelligence scale for children (WISC); (2) the wide range achievement test (WRAT); (3) the NCPP behavioral checklist	(1) IQ ≥ 80 on WISC; (2) reading scores < 1.5 SD on WRAT; (3) in the first or second grade at the time of testing; (4) English was the primary language; (5) score was normal on the NCPP behavioral checklist	Total = 32,223 boys = 16,080 girls = 16,143	Total = 1410 boys = 947 girls = 463	Total = 4.38% boys = 5.89% girls = 2.87%
13	Lv et al., 2000	China	random sampling	logographic script	Western Pacific	MIC	(1) A self-compiled children’s family environment questionnaire; (2) the Wechsler intelligence scale for children (WISC)	(1) IQ > 70; (2) 1 SD below the average score of their peers in one or more subjects; (3) equal learning opportunities with other children; (4) no nervous system diseases, visual, auditory, or motor disorders	Total = 688 boys = 357 girls = 331	Total = 65 boys = 44 girls = 21	Total = 9.45% boys = 12.32% girls = 6.34%
14	Bhakta et al., 2002	India	stratified random sampling	alphabetic script	South-East Asia	MIC	(1) The Malayalam translation of the Rutter A2 parent-completed scale; (2) the Malayalam graded reading test (MGRT); (3) the Malayalam vocabulary test (MVT); (4) Raven’s colored progressive matrices, (CPM); (5) the short-form Oseretsky test of motor proficiency; 6) the Rutter B2 teacher-completed scale (Malayalam version)	A GMRT score of less than 20	Total = 119 boys = 604 girls = 566	Total = 98 boys = 71 girls = 27	Total = 8.22% boys = 11.75% girls = 4.77%
15	Yao et al., 2003	China	random sampling	logographic script	Western Pacific	MIC	(1) The pupil rating scale–revised screening for learning disabilities (PRS); (2) Conners parent symptom questionnaire (PSQ); (3) the YG personality scale; (4) a self-compiled questionnaire on the general conditions of parents and children	(1) A score of PRS < 60 (2) IQ > 80; (3) No history of congenital diseases and traumatic brain injury.	Total = 1151 boys = 605 girls = 546	Total = 118 boys = 79 girls = 39	Total = 10.25% boys = 13.06% girls = 7.14%
16	Han Juan, 2005	China	random sampling	logographic script	Western Pacific	MIC	(1) The pupil rating scale–revised screening for learning disabilities (PRS); (2) general situation questionnaire; (3) Conners parent symptom questionnaire (PSQ); (4) revised children’s self-concept scale (PHCSS); (5) Wechsler intelligence scale for children–Chinese revision (WISC-CR); (6) Wechsler memory scale (WMS); (7) digital cancellation, digital connection test A and word fluency test; (8) children’s sensory integration development rating scale	(1) A score of PRS ≤ 60; (2) the average score of the main course (Chinese, mathematics) was below the 10 percentile of the class, with LD lasting more than one year, and it was considered difficult to complete the class and homework independently; (3) the reading test score was less than 1 SD of the mean of group test scores; (4) IQ ≥ 85; (5) no motivational problems, attention deficit hyperactivity disorder, emotional disorders and other psychological problems, no organic encephalopathy	Total = 800	Total = 65	Total = 8.13%
17	Pan et al., 2006	China	random sampling	logographic script	Western Pacific	MIC	(1) IQ self-test; (2) learning disability behavior scale; (3) the learning motivation diagnostic test (MAAT); (4) the enhanced learning factor diagnostic test (FAT)	(1) The IQ score was between 85 and 140; (2) there were one or more cases of I value ≥ 24, II value ≥ 18, III value ≥ 21, IV value ≥ 9, V value ≥ 18, VI value ≥ 12, VII value ≥ 12 in the LD behavior scale	Total = 332 boys = 169 girls = 161 3rd = 164 5th = 168	Total = 50 boys = 28 girls = 22 3rd = 27 5th = 23	Total = 15.06% boys = 16.57% girls = 13.66% 3rd = 16.46% 5th = 13.69%
18	Song Ranran, 2006	China	random sampling	logographic script	Western Pacific	MIC	(1) A family situation questionnaire compiled by the Shanghai Mental Health Center; (2) the pupil rating scale–revised screening for learning disabilities (PRS); (3) the dyslexia checklist for Chinese (DCCC); (4) the Wechsler intelligence scale for children–Chinese revision (WISC-CR)	(1) A score of PRS ≤ 60; (2) academic performance was in the bottom 10%; (3) the DCCC score was less than 2 SD of students in the same grade; (4) an IQ > 80 and no visual, auditory impairment, no organic lesions	Total = 1096 boys = 589 girls = 507 3rd = 533 4th = 370 5th = 193	Total = 69 boys = 49 girls = 20 3rd = 36 4th = 22 5th = 11	Total = 6.30% boys = 8.32% girls = 3.94% 3rd = 6.75% 4th = 5.95% 5th = 5.70%
19	Yu Yizhen, 2006	China	random sampling	logographic script	Western Pacific	MIC	(1) The pupil rating scale–revised screening for learning disabilities (PRS); (2) Chinese classification and diagnostic criteria of mental disorders (2nd edition) (CCMD-2-R); (3) the second revision of the Chinese combined Raven’s test (CRT-C2); (4) a general situation questionnaire	(1) A score of PRS ≤ 60; (2) meeting the standard of LD in CCMD-2-R; (3) the average score of the main course (Chinese, Mathematics) was below the 10 percentile of the class, and it was difficult to complete the class and homework independently; (4) IQ > 70; (5) no visual or hearing impairment, no hyperactivity and organic lesions	Total = 903 boys = 496 girls = 407	Total = 90 boys = 58 girls = 32	Total = 9.97% boys = 11.69% girls = 7.86%
20	Chan et al., 2007	China	stratified random sampling	logographic script	Western Pacific	HIC	(1) The Hong Kong test of specific learning difficulties in reading and writing (HKT-SpLD); (2) the Hong Kong Wechsler intelligence scale for children (HK-WISC)	(1) Scoring 7 or less on the literacy test domain and on one or more of the cognitive test domains; (2) IQ ≥ 85	Total = 690 boys = 350 girls = 340	Total = 67 boys = 45 girls = 22	Total = 9.71% boys = 12.86% girls = 6.47%
21	Lu Shan, 2007	China	random sampling	logographic script	Western Pacific	MIC	(1) A general situation questionnaire; (2) the pupil rating scale–revised screening for learning disabilities (PRS); (3) the second revision of the Chinese combined Raven’s test (CRT-C2); (4) the dyslexia checklist for Chinese (DCCC)	(1) A score of PRS < 65; (2) the Chinese score lags behind the average score of the same class by more than 1 SD, with LD lasting more than one year, and it was difficult to complete the class and homework independently; (3) the reading test score was less than 2 SD of the mean of group test scores; (4) IQ > 70; (5) excluding other disabilities and environmental factors	Total = 820 boys = 427 girls = 393 3rd = 332 4th = 213 5th = 275	Total = 55 boys = 43 girls = 12 3rd = 23 4th = 15 5th = 17	Total = 6.70% boys = 10.07% girls = 3.05% 3rd = 6.93% 4th = 7.04% 5th = 6.18%
22	Fluss et al., 2008	France	stratified sampling	alphabetic script	Europe	HIC	(1) Reading comprehension; (2) spelling skill; (3) mathematical knowledge	On reading/spelling/mathematics (FL, FO, FM, respectively), children’ scores were below 1 SD	Total = 1020 boys = 544 girls = 476	Total = 130	Total = 12.70%
23	Wang Zhong, 2008	China	stratified sampling	logographic script	Western Pacific	MIC	(1) The pupil rating scale–revised screening for learning disabilities (PRS); (2) the combined Raven’s test (CRT)	According to ICD-10, the total score of PRS was less than 60, or the score of verbal type (factor A and B) was less than 20, or the score of non-verbal type (factor C, D and E) was less than 40	Total = 3934 boys = 2321 girls = 1613 1st = 601 2nd = 617 3rd = 668 4th = 689 5th = 669 6th = 690	Total = 407 boys = 326 girls = 81 1st = 87 2nd = 63 3rd = 69 4th = 71 5th = 60 6th = 57	Total = 10.35% boys = 14.05% girls = 5.02% 1st = 14.48% 2nd = 10.21% 3rd = 10.33% 4th = 10.30% 5th = 8.97% 6th = 8.26%
24	Zou Yuliang, 2008	China	random sampling	logographic script	Western Pacific	MIC	(1) The dyslexia checklist for Chinese (DCCC); (2) The second revision of the Chinese combined Raven’s test (CRT-C2); (3) a students’ family situation questionnaire compiled by the research group	(1) T scores of each factor or the whole score of DCCC scale were above 98 percentile points; (2) IQ > 80	Total = 255 boys = 123 girls = 132	Total = 25 boys = 19 girls = 6	Total = 9.80% boys = 15.45% girls = 4.55%
25	Shaheen, H. A., 2010	Egypt	random sampling	alphabetic script	Eastern Mediterranean	MIC	Arabic reading tests (ART)	(1) With no visual, hearing problems, motor impairment, mental retardation (IQ less than 90%) or major psychological disorder; (2) scored 40 or less in ART	Total = 206 boys = 117 girls = 89	Total = 22 boys = 12 girls = 10	Total = 10.68% boys = 10.26% girls = 11.24%
26	Zou et al., 2010	China	random sampling	logographic script	Western Pacific	MIC	(1) A family reading environment and reading ability questionnaire; (2) the dyslexia checklist for Chinese (DCCC); (3) the pupil rating scale–revised screening for learning disabilities (PRS); (4) the second revision of the Chinese combined Raven’s test (CRT-C2)	(1) The total score of DCCC was more than 2 SD higher than the mean score; (2) a score of PRS < 65; (3) academic achievement was at the bottom 10% of the class; (4) IQ > 80; (5) no visual, auditory impairment, no organic lesions	Total = 587 boys = 305 girls = 282	Total = 23 boys = 18 girls = 5	Total = 3.92% boys = 5.90% girls = 1.77%
27	Daseking et al., 2011	Germany	random sampling	alphabetic script	Europe	HIC	The social–paediatric screening of developmental status for school entry (SOPESS)	A PR of no more than 10 on the SOPESS	Total = 372	Total = 11	Total = 2.96%
28	Jiménez et al., 2011 (study 1)	Spain	random sampling	alphabetic script	Europe	HIC	(1) Culture-fair (or -free) intelligence tests; (2) reading comprehension test; (3) fluency task; (4) working memory test	(1) No absence of sensory, acquired neurological and other problems; (2) a percentile score below 25 on accuracy on pseudoword reading from the naming task, or a percentile above 75 on reading time on pseudoword or word reading from the naming task; (3) IQ > 75	Total = 1048 boys = 630 girls = 418	Total = 164 boys = 98 girls = 66	Total = 15.65% boys = 15.56% girls = 15.79%
28	Jiménez et al., 2011 (study 2)	Guatemalan	random sampling	alphabetic script	Americas	MIC	(1) Culture-fair (or -free) intelligence tests; (2) reading comprehension test; (3) fluency task; (4) working memory test	(1) No absence of sensory, acquired neurological and other problems; (2) a percentile score below 25 on accuracy on pseudoword reading from the naming task, or a percentile above 75 on reading time on pseudoword or word reading from the naming task; (3) IQ > 75	Total = 557 boys = 316 girls = 241	Total = 110 boys = 65 girls = 45	Total = 19.90% boys = 20.57% girls = 18.67%
29	Pouretemad et al., 2011	Iran	random sampling	alphabetic script	Eastern Mediterranean	MIC	(1) An analysis of Persian reading ability (APRA); (2) Wechsler intelligence scale for children–third edition (WISC-III)	(1) IQ ≥ 85; (2) reading scores in three trimesters of one academic year were more than 1.5 SD below that expected from their math scores; (3) no history of brain damage, hearing or visual problems	Total = 1562 boys = 773 girls = 789 1st = 298 2nd = 271 3rd = 309 4th = 330 5th = 354	Total = 82 boys = 59 girls = 23 1st = 11 2nd = 9 3rd = 22 4th = 20 5th = 20	Total = 5.20% boys = 7.63% girls = 2.92% 1st = 3.69% 2nd = 3.32% 3rd = 7.12% 4th = 6.06% 5th = 5.65%
30	Vale et al., 2011	Portugal	random sampling	alphabetic script	Europe	HIC	(1) The TIL-reading age test; (2) the PRP–word recognition test; (3) the MPC Raven; (4) the phonological awareness tests of the ALEPE battery	(1) Achieved a result equal to or less than the percentage 5 in the TIL; (2) a result below the PRP mastery criteria; (3) normal IQ; (4) the phonological awareness score was significantly lower than those presented by control groups	Total = 1360 2nd = 493 3rd = 445 4th = 422	Total = 74 boys = 45 girls = 29 2nd = 38 3rd = 15 4th = 21	Total = 5.44% 2nd = 7.70% 3rd = 3.37% 4th = 4.98%
31	Zhu Dongmei, 2011	China	random sampling	logographic script	Western Pacific	MIC	(1) A general situation questionnaire; (2) the pupil rating scale–revised screening for learning disabilities (PRS); (3) the dyslexia checklist for Chinese (DCCC); (4) Chinese reading ability test; (5) the second revision of the Chinese combined Raven’s test (CRT-C2)	(1) A score of PRS < 65; (2) Chinese scores were in the bottom 10 of the class. According to the head teacher’s evaluation, they had learning difficulties lasting more than one year, and had difficulties in completing the classroom and homework independently; (3) IQ > 80; (4) the converted T-score of DCCC was lower than the mean plus 2 SD; (5) scores 2 SD below the standard score on Chinese reading ability test; 6) no other diseases and environmental factors	Total = 1048 boys = 513 girls = 535 3rd = 425 4th = 426 5th = 197	Total = 74 Boy = 44 girls = 30 3rd = 37 4th = 20 5th = 17	Total = 7.10% boys = 8.6% girls = 5.6% 3rd = 8.7% 4th = 4.7% 5th = 8.6%
32	Mogasale et al., 2012	India	stratified random sampling	alphabetic script	South-East Asia	MIC	(1) Rutter‘s proforma A; (2) Seguin form board test; (3) the specific learning disabilities (SpLD) battery test	(1) Poor grades (C or C+) of academic record in two consecutive examinations; (2) no visual, hearing disorders or severe physical conditions; (3) IQ ≥ 90	Total = 1079	Total = 121	Total = 11.21%
33	Luo Yan, 2012	China	random sampling	logographic script	Western Pacific	MIC	(1) The dyslexia checklist for Chinese (DCCC); (2) The pupil rating scale–revised screening for learning disabilities (PRS); (3) the second revision of the Chinese combined Raven’s test (CRT-C2)	(1) The transformed T-scord of DCCC > 70; (2) a score of PRS < 65; (3) Chinese score ranked in the bottom 10 of the class, with LD lasting more than one year, and it was difficult to complete the class and homework independently; (4) IQ ≥ 80; (5) no visual, auditory impairment, no organic lesions	Total = 435 boys = 221 girls = 214 3rd = 136 4th = 159 5th = 140	Total = 33 boys = 23 girls = 10 3rd = 12 4th = 10 5th = 11	Total = 7.59% boys = 10.41% girls = 4.68% 3rd = 8.82% 4th = 6.29% 5th = 7.86%
34	Zhao Xiaochen, 2013	China	random sampling	logographic script	Western Pacific	MIC	(1) The Hong Kong behavior checklist of specific learning difficulties in reading and writing for primary school students (second edition) (BCL-P(II)); (2) Conners’ teacher rating scale; (3) Raven’s test; (4) the Hong Kong-specific learning difficulties behavior checklist (HKSLDBC); (5) the Hong Kong test of specific learning difficulties in reading and writing (HKT-SpLD)	(1) The students in the bottom 25% of each grade were selected according to their most recent grade scores in Chinese and math; (2) the score on the BCL scale was greater than or equal to 18; (3) IQ ≥85; (4) subjects performed 1 SD lower than the average level of the same grade in one-minute word reading task, Chinese word reading task, literacy task, and fast naming task; (5) no brain injury, emotional or behavioral problems	Total = 1069	Total = 49	Total = 4.58%
35	Zuo et al., 2013	China	random sampling	logographic script	Western Pacific	MIC	(1) The pupil rating scale–revised screening for learning disabilities (PRS); (2) the dyslexia checklist for Chinese, (DCCC); (3) the Wechsler intelligence scale for children–Chinese revision (WISC-CR)	(1) A score of PRS < 65; (2) the DCCC score was lower than the standard score by 2 SD; (3) IQ > 70; (4) no visual or auditory impairment, no organic lesions	Total = 1206 boys = 621 girls = 585 3rd = 401 4th = 398 5th = 409	Total = 82 boys = 55 girls = 27 3rd = 27 4th = 26 5th = 31	Total = 6.80% boys = 8.86% girls = 4.62% 3rd = 6.73% 4th = 6.53% 5th = 7.58%
36	Liu et al., 2014	China	random sampling	logographic script	Western Pacific	MIC	(1) The one-minute Chinese word reading test; (2) Raven’s standard progressive matrices (SPM)	(1) The Chinese teachers in the bilingual classes of each grade selected the bottom 10 students in the class, based on the children’s Chinese test scores; (2) the 10 students tested the self-compiled “One-minute Chinese Word Reading Test”, and then selected children whose scores were lower than the percentile grade corresponding to 1.5 SD from the average score of the grade norm; (3) no obvious physiological injury, behavioral and emotional disorders; (4) Raven percentile level above 25% on SPM	Total = 1397 3rd = 458 4th = 418 5th = 521	Total = 46 3rd = 15 4th = 11 5th = 20	Total = 3.29% 3rd = 3.28% 4th = 2.63% 5th = 3.84%
37	Irene Jepkoech Cheruiyot, 2015	The Republic of Kenya	random sampling	alphabetic script	Africa	MIC	(1) The Burt reading test (1974) revised; (2) the Pearson dyslexia screening test for juniors (DST-J); (3) a socio-demographic questionnaire	(1) Reading age was way below chronological age (by 9 months or more) on the Burt reading test (1974)–revised; (2) an at-risk quotient of 0.6 or greater on the DST-J	Total = 120 boys = 63 girls = 57	Total = 9 boys = 6 girls = 3	Total = 7.50% boys = 9.52% girls = 5.26%
38	Liu et al., 2016	China	random sampling	logographic script	Western Pacific	MIC	(1) The dyslexia checklist for Chinese children (DCCC); (2) the pupil rating scale–revised screening for learning disabilities (PRS)	(1) The score of DCCC was 2 SD higher than the mean score of all the students in the same grade; (2) a score of PRS < 65; (3) the Chinese language exam was below the 10% of all children in the same grade; (4) no intellectual disability, brain injury, visual and auditory disorders, epilepsy, or other neurological disorders.	Total = 34,748 boys = 16,752 girls = 16,645 3rd = 7901 4th = 8387 5th = 8591 6th = 8669	Total = 1200 boys = 893 girls = 301 3rd = 316 4th = 332 5th = 297 6th = 255	Total = 3.45% boys = 5.06% girls = 1.78% 3rd = 3.85% 4th = 3.81% 5th = 3.34% 6th = 2.86%
39	Padhy et al., 2016	India	stratified random sampling	alphabetic script	South-East Asia	MIC	(1) The specific learning disability screening questionnaire (SLD-SQ); (2) Brigance diagnostic inventory (BDI)—part of NIMHANS index of specific learning disabilities	(1) Being considered by the teacher to have some form of learning difficulty; (2) scored above 4 on the SLD-SQ	Total = 3600	Total = 108	Total = 3.08%
40	Sheikh et al., 2016	Egypt	stratified random sampling	alphabetic script	Eastern Mediterranean	MIC	(1) The reading disability test (RDT); (2) the Wechsler intelligence scale for children (WISC); (3) the “kiddie“ schedule for affective disorders and schizophrenia, present and lifetime versions (k-SADSPL)	Students whose reading scores were below the cut-off score (57 for fifth grade, 49 for fourth grade) of RDT and IQ levels of 90 or more	Total = 567 boys = 305 girls = 262	Total = 64 boys = 37 girls = 27	Total = 11.30% boys = 12.13% girls = 10.31%
41	Song Yi, 2016	China	random sampling	logographic script	Western Pacific	MIC	(1) The pupil rating scale–revised screening for learning disabilities (PRS); (2) the second revision of the Chinese combined Raven’s test (CRT-C2); (3) the dyslexia checklist for Chinese (DCCC)	(1) The Chinese score was ranked in the bottom 15% of the grade; (2) the language part of the PRS scale scored less than 20 points; (3) normal IQ; (4) the transformed T-score of DCCC > 70; (5) no visual, auditory and other sensory disorders, no nervous system diseases	Total = 395 boys = 200 girls = 195	Total = 23 boys = 16 girls = 7	Total = 5.80% boys = 8.00% girls = 3.59%
42	Zhang et al., 2016	China	stratified sampling	logographic script	Western Pacific	MIC	(1) A family economic environment and reading ability questionnaire; (2) the dyslexia checklist for Uygur children (DCUC); (3) the Wechsler intelligence scale for children–Chinese revision (WISC-CR)	(1) The transformed T-scored of DCUC > 70; (2) IQ > 80; (3) no visual, auditory impairment, no organic lesions	Total = 3508 boys = 1837 girls = 1671 3rd = 1281 4th = 1210 5th = 1017	Total = 207 boys = 144 girls = 63 3rd = 85 4th = 75 5th = 47	Total = 5.90% boys = 7.84% girls = 3.78% 3rd = 6.63% 4th = 6.20% 5th = 4.62%
43	Zhao et al., 2016	China	stratified sampling	logographic script	Western Pacific	MIC	(1) The pupil rating scale–revised screening for learning disabilities (PRS); (2) the dyslexia checklist for Chinese children (DCCC); (3) the dyslexia checklist for Uyghur children (DCUC); (4) the home literacy environment and reading ability survey scale (HLE-RA); (5) the China–Wechsler intelligence scale for children (C-WISC)	(1) A score of PRS < 65; (2) the score of DCCC was 2 SD higher than the mean scores of Han Chinese children; DCUC score was 2 SD higher than the mean scores of Uyghur children; (3) IQ > 80; (4) no visual and/or auditory disorders or psychiatric diseases	Total = 2348 boys = 1163 girls = 1185 3rd = 623 4th = 719 5th = 798 6th = 208	Total = 129 boys = 86 girls = 43 3rd = 39 4th = 48 5th = 39 6th = 3	Total = 5.49% boys = 7.39% girls = 3.63% 3rd = 6.26% 4th = 6.68% 5th = 4.89% 6th = 1.44%
44	Cuadro et al., 2017	Spain	stratified sampling	alphabetic script	Europe	HIC	(1) Reading efficiency test; (2) orthographic level test	A cut-off point of 1.5 SD below the mean of each school year in the reading efficiency test	Total = 1408 boys = 718 girls = 690 2nd = 308 3rd = 305 4th = 273 5th = 271 6th = 251	Total = 75 boys = 47 girls = 28 2nd = 10 3rd = 12 4th = 12 5th = 22 6th = 19	Total = 5.32% boys = 6.55% girls = 4.06% 2nd = 3.20% 3rd = 3.90% 4th = 4.40% 5th = 8.10% 6th = 7.60%
45	Qian Lizhu, 2017	China	random sampling	Chinese	Western Pacific	MIC	The dyslexia checklist for Chinese children (DCCC)	T score of any factor or full scale ≥ 70	Total = 325 boys = 179 girls = 146 5th = 221 6th = 104	Total = 38 boys = 29 girls = 9 5th = 26 6th = 12	Total = 11.69% boys = 16.20% girls = 6.16% 5th = 11.76% 6th = 11.54%
46	Wang Rui, 2017	China	random sampling	logographic script	Western Pacific	MIC	(1) Chinese character literacy test for primary school students; (2) the pupil rating scale–revised screening for learning disabilities (PRS); (3) Raven’s standard progressive matrices (SPM); (4) the grade of Chinese	(1) The literacy level was 1.5 SD below the grade average, according to the Chinese character literacy test for primary school students; (2) a score of PRS < 65; (3) normal IQ; (4) The students’ Chinese score was lower than the grade average level in the past half a year	Total = 847	Total = 66	Total = 7.79%
47	Yan Nairui, 2018	China	random sampling	logographic script	Western Pacific	MIC	(1) A parental rearing style assessment scale (EMBU); (2) the family environment scale (EFS); (3) a self-compiled specific learning disability screening questionnaire; (4) a self-compiled children’s mental development assessment questionnaire; (5) a self-compiled questionnaire on the risk factors of pregnancy, lactation and early childhood	(1) The students in the bottom 25% of each grade were selected according to their most recent grade scores in Chinese and math; (2) a score of the specific learning disability screening questionnaire ≥ 34	Total = 1179 boys = 642 girls = 537 1st = 382 3rd = 465 5th = 332	Total = 139 boys = 92 girls = 47 1st = 46 3rd = 55 5th = 38	Total = 11.79% boys = 14.33% girls = 8.75% 1st = 12.04% 5th = 11.45% 3rd = 11.83%
48	Yoo et al., 2018	South Korea	random sampling	alphabetic script	Western Pacific	MIC	(1) The dyslexia screening checklist (DySC); (2) Korean–Wechsler intelligence scale for children—fourth edition (K-WISC-IV); (3) the comprehensive learning test–reading (CLT-R); (4) the comprehensive learning test–math (CLT-M); (5) the comprehensive attention test (CAT)	Being in the bottom 15% on DySC and CLT-R, and having no intelligence or attention problems	Total = 659 boys = 340 girls = 319	Total = 37 boys = 22 girls = 15	Total = 5.61% boys = 6.473% girls = 4.70%
49	Zhou et al., 2018	China	random sampling	logographic script	Western Pacific	MIC	(1) The dyslexia checklist for Chinese (DCCC); (2) the second revision of the Chinese combined Raven’s test (CRT-C2); (3) the pupil rating scale–revised screening for learning disabilities (PRS)	(1) The transformed T-scored of DCCC > 70; (2) the Chinese score ranked in the bottom 10 of the class, with LD lasting more than one year, and it was difficult to complete the class and homework independently; (3) a score of PRS > 65; (4) IQ ≥ 80; (5) no visual, auditory and other sensory disorders, no nervous system diseases	Total = 369 boys = 188 girls = 181	Total = 15 boys = 13 girls = 2	Total = 4.07% boys = 6.9% girls = 1.1%
50	Barbiero et al., 2019	Italy	random sampling	alphabetic script	Europe	HIC	(1) A questionnaire derived from the validated questionnaire “RSR-DSA”; (2) a 4th-grade dictation task; (3) the DDE-2 battery (battery for the assessment of developmental dyslexia and dysorthographia-2); (4) the Wechsler intelligence scale for children (WISC-III); (5) battery for the evaluation of developmental dyslexia and dysorthography-2 (DDE-2); (6) the MT battery (prove di lettura MT per la scuola elementare-2); (7) Raven’s progressive matrices (PM47); (8) a strengths and difficulties questionnaire (SDQ)	(1) The total score was > 85% or the score on two subgroups of questions specifically addressing dyslexia > 90%; (2) children scoring ≥ 90% in the dictation task; (3) children failed in at least one of four scores in DDE-2; (4) WISC-III weighted score > 7; (5) Z-score ≤ −1.8 (speed) or percentile ≤ 5 (accuracy) in the DDE-2 non-word test	Total = 9964	Total = 350	Total = 3.51%
51	Fan et al., 2019	China	random sampling	Chinese	Western Pacific	MIC	Multiple achievement tests (MATs)	(1) The scores of the last three Chinese mid-term and final exams were lower than the grade average level and the math scores were normal; (2) the evaluation results of Chinese teachers on students’ Chinese reading performance; (3) no brain damage or intellectual, visual or hearing impairment; (4) students scored 1.5 SD below the norm on standardized reading tests	Total = 834 boys = 444 girls = 390	Total = 62 4th = 35 5th = 27	Total = 7.43%
52	Gu et al. 2019	China	Stratified cluster sampling	Chinese	Western Pacific	MIC	(1) The dyslexia checklist for Chinese children (DCCC); (2) the pupil rating scale–revised screening for learning disabilities (PRS);	(1) No brain diseases such as visual and hearing impairment, brain trauma, epilepsy, etc.; (2) the Chinese score was in the last 10% of the class; (3) one subscale or total score in the DCCC was 2 SD higher than that of children of the same age; (4) the score of the PRS was < 65	Total = 11,668 boys = 6289 girls = 5369 2nd = 2916 3rd = 2743 4th = 2254 5th = 2537 6th = 1218	Total = 302 boys = 233 girls = 69 2nd = 79 3rd = 66 4th = 58 5th = 665 6th = 33	Total = 2.59% boys = 3.7% girls = 1.29% 2nd = 2.71% 3rd = 2.41% 4th = 2.57% 5th = 2.60% 6th = 2.71%
53	Zhu et al., 2019	China	Stratified cluster sampling	Chinese	Western Pacific	MIC	(1) The dyslexia checklist for Chinese children (DCCC); (2) the pupil rating scale–revised screening for learning disabilities (PRS);	(1) No brain diseases such as visual and hearing impairment, brain trauma, epilepsy, etc.; (2) the Chinese score was in the last 10% of the class; (3) one subscale or total score in the DCCC was 2 SD higher than that of children of the same age; (4) score of the PRS < 65	Total= 3673 boys= 2118 girls= 1555 3rd= 838 4th= 924 5th = 946 6th = 965	Total= 119 boys= 95 girls= 24 3rd= 13 4th= 29 5th = 36 6th = 41	Total= 3.24% boys= 4.49% girls= 1.54% 3rd= 1.55% 4th= 3.14% 5th= 3.81% 6th= 4.25%
54	Cai et al., 2020	China	Stratified cluster sampling	Chinese	Western Pacific	MIC	(1) The pupil rating scale–revised screening for learning disabilities (PRS); (2) the Chinese character recognition measure and assessment scale for primary school children; (3) a combined Raven’s test	(1) PRS score below 65; (2) at least 1 SD below the average level of actual grade in Chinese character recognition; (3) IQ > 80; (4) according to the head-teachers’ reports, there was no suspected brain damage, uncorrected sensory impairment, or other external factors	Total = 1661 boys = 882 girls = 779 2nd = 452 3rd = 407 4th = 432 5th = 370	Total = 81 boys = 66 girls = 15 2nd = 28 3rd = 13 4th = 24 5th = 16	Total = 4.88% boys = 7.48% girls = 1.93% 2nd = 6.19% 3rd = 3.19% 4th = 5.56% 5th = 4.32%
55	Su et al., 2020	China	Random sampling	Chinese	Western Pacific	MIC	Raven’s standard progressive matrices (SPM)	(1) The Chinese score was at the bottom 10% of the class; (2) an IQ score of above 25 percent on the SPM test; (3) no hearing impairment, attention deficit, hyperactivity disorder, autism or mood disorders	Total = 624 3rd = 217 4th = 224 5th = 183	Total = 62 3rd = 22 4th = 22 5th = 18	Total= 9.94% 3rd = 10.14% 4th = 9.82% 5th = 9.84%
56	YILIZHATI et al., 2020	China	Random sampling	Chinese	Western Pacific	MIC	(1) One-minute word reading test; (2) Raven’s intelligence test	(1) Students whose reading level was considered by the teacher to be at the bottom 25% of the class; (2) the score of “one-minute word reading test” was 1 SD lower than the grade average; (3) no obvious physical injury, behavioral and emotional disorders; (4) an IQ score of above 25 percent on the SPM test	Total = 1233	Total = 119	Total = 9.65%
